# Importance of physical detraining in functional capacity of individuals with chronic peripheral arterial occlusive disease: a cross-sectional pilot study

**DOI:** 10.1590/1677-5449.200237

**Published:** 2021-05-10

**Authors:** Mabel Marciela Ahner, Adamastor Humberto Pereira, Alexandre Araújo Pereira, Gabriel Alves Fonseca, Gabriel Pereira dos Reis Zubaran, Débora dos Santos Macedo, Eduardo Lima Garcia, Leandro Tolfo Franzoni

**Affiliations:** 1 Universidade Federal do Rio Grande do Sul – UFRGS, Hospital de Clínicas de Porto Alegre – HCPA, Porto Alegre, RS, Brasil.; 2 Universidade Federal do Rio Grande do Sul – UFRGS, Hospital de Clínicas de Porto Alegre – HCPA, Ambulatório de Cirurgia Vascular, Porto Alegre, RS, Brasil.; 3 Universidade Federal do Rio Grande do Sul – UFRGS, Programa de Pós-graduação em Ciências da Saúde: Cardiologia e Ciências Cardiovasculares, Porto Alegre, RS, Brasil.

**Keywords:** exercise, peripheral arterial disease, intermittent claudication

## Abstract

**Background:**

Physical training is a well-established strategy for rehabilitation of the functional capacity of individuals with chronic peripheral arterial occlusive disease (PAOD). However, some individuals quit training after participating in a physical training program, undergoing detraining. There is scant literature on the effects of physical detraining in individuals with PAOD and it is therefore important to investigate the effects of this phenomenon.

**Objectives:**

The objective of this article was to evaluate the effects of physical detraining on functional capacity in individuals with PAOD.

**Methods:**

Cross-sectional study with 22 individuals. Participants were divided into two groups: a detraining group (DG) and a control group (CG). The distance covered in the 6-minute walk test (6MWTD) and the pain-free walking distance (PFWD) were evaluated. The PFWD is the distance covered until claudication begins, i.e., the distance covered without pain.

**Results:**

Mean age was 66 ± 8 in the DG and 67 ± 7 in the CG. There were no differences between the groups in either the 6MWTD or the PFWD (p = 0.428; p = 0.537, respectively).

**Conclusions:**

The present pilot study allows us to conclude that the functional capacity of individuals with PAOD who participated in a physical training program and subsequently underwent detraining was not superior in relation to individuals who did not participate in a physical training program. The results of the present study serve to encourage maintenance of physical exercise, since physical training is no longer effective if detraining occurs.

## INTRODUCTION

Functional capacity is reduced in chronic peripheral arterial occlusive disease (PAOD) because of the atherosclerotic process involving the peripheral arteries,^1^ with consequent obstruction of the blood flow to the lower limbs, provoking the classic symptom known as intermittent claudication.^2^ The shorter the distance an individual can walk before claudication sets in, i.e. free from pain, the worse the disease prognosis.^3^

Physical training is one of the most important tools for increasing the functional capacity of people with PAOD.^4^ The distance covered in a 6-minute walk test (6MWTD) and the claudicant pain-free walking distance (PFWD) are important outcomes related to functional capacity.^3^^,^ 5 Several different studies have compared the effects of physical training on the 6MWTD and PFWD, whether aerobic exercises, strength training, or a combination of the two, and with durations ranging from 8 to 16 weeks.^6^^-^ 10 However, after the intervention period, patients tend to give up on physical training.

Physical detraining raises questions about maintenance of the effects yielded by physical training, in terms of the 6MWTD and PFWD achieved by patients with PAOD. However, there is a gap in the literature, which lacks studies investigating the effects of physical detraining in PAOD.^11^^-^ 13 In view of this, the objective of the present study was to compare the effects of physical detraining on 6MWTD and PFWD in patients with PAOD. The hypothesis raised is that individuals who participated in a physical training program but then underwent detraining would exhibit greater benefit than individuals who had not participated in the physical training program.

## METHODS

### Study design

This is a cross-sectional, case-control study. The participants were divided into two groups: a detraining group (DG), comprising individuals who had participated in a 12-week physical training program and then undergone detraining for at least 3 months up to a maximum of 36 months; and a control group (CG), comprising patients with PAOD who had not participated in a physical training program in the preceding 36 months.

### Study setting and participants

The study was conducted at the Vascular Surgery Outpatient Clinic of a university hospital in southern Brazil, in 2018 and 2019. Patients over the age of 40 years with a diagnosis of intermittent claudication were recruited. All participants should also have an ankle-brachial index (ABI) below 0.9. The exclusion criteria were: cardiovascular events that occurred less than 3 months before enrollment; uncontrolled severe hypertension (systolic blood pressure ≥ 180 mmHg or diastolic blood pressure ≥ 110 mmHg) and/or uncontrolled diabetes (glycemic index ≥ 290); critical lower limb ischemia; limiting lung disease; or any type of contraindication to the exercise test.

The study was approved by the local Research Ethics Committee (decision number 3.240.172) and conducted entirely in accordance with the ethical standards set out in the Helsinki Declaration. All patients provided written consent to participation before enrollment. One of the study groups (DG) comprised patients who had taken part in a randomized clinical trial that investigated the effects of 12 weeks of physical training on the functional capacity of individuals with PAOD.

### Experimental procedures

#### Functional capacity – 6MWTD and PFWD

Walking capacity was measured using the 6-minute walk test (6MWT) by a group of specialists, following American Thoracic Society guidelines.^14^ During the 6MWT, we used a visual analog scale (VAS)^15^^,^ 16 to measure pain in the lower limbs, and also administered the Borg subjective exertion scale.^17^ The test was conducted in a 30m corridor and patients were instructed to walk as far as possible in 6 minutes. Blood pressure and heart rate were measured before and after the test. Patients were asked about onset of pain and instructed to walk as rapidly as possible. The distance walked before onset of pain and the total distance walked were noted and expressed in meters.

### Physical activity level

For the purposes of the present study, a simple questionnaire was administered covering physical activity levels. This instrument was based on the short form of the International Physical Activity Questionnaire (IPAQ).^18^ Patients were questioned about the number of days per week and the length of time, in minutes, for which they performed mild, moderate, and vigorous walking.

### Possible biases

The sample was rigorously selected to minimize possible biases, with full patient history and clinical examination conducted by the team from the Lymphedema and Angiodysplasia Clinic. Additionally, all individuals who performed the 6MWT were assessed by the same researcher, to eliminate intra-examiner variability, even though a protocol recommended in the literature was followed.^14^

### Statistical analysis

Sample size was calculated to detect differences between groups of 60 meters (m) for 6MWTD and 30 m for PFWD, with a standard deviation equal to the 6MWTD difference to be detected, 80% power, and a 5% significance level, which resulted in 14 participants per group.^7^

The Shapiro Wilk and Levene tests were used to analyze normality and homogeneity of data respectively. Student’s *t* test (unpaired) or the Mann-Whitney U test were used to analyze differences between groups for the main outcomes (6MWTD and PFWD) and other continuous variables, and the chi-square test was used for categorical variables. Normally distributed data were expressed as means and standard deviations and non-normal data were expressed as medians and interquartile ranges. Data were analyzed using SPSS, version 20.0 (IBM, Corporation, Armonk, NY, United States). Results with p < 0.05 were considered statistically significant.

## RESULTS

**Figure 1:**
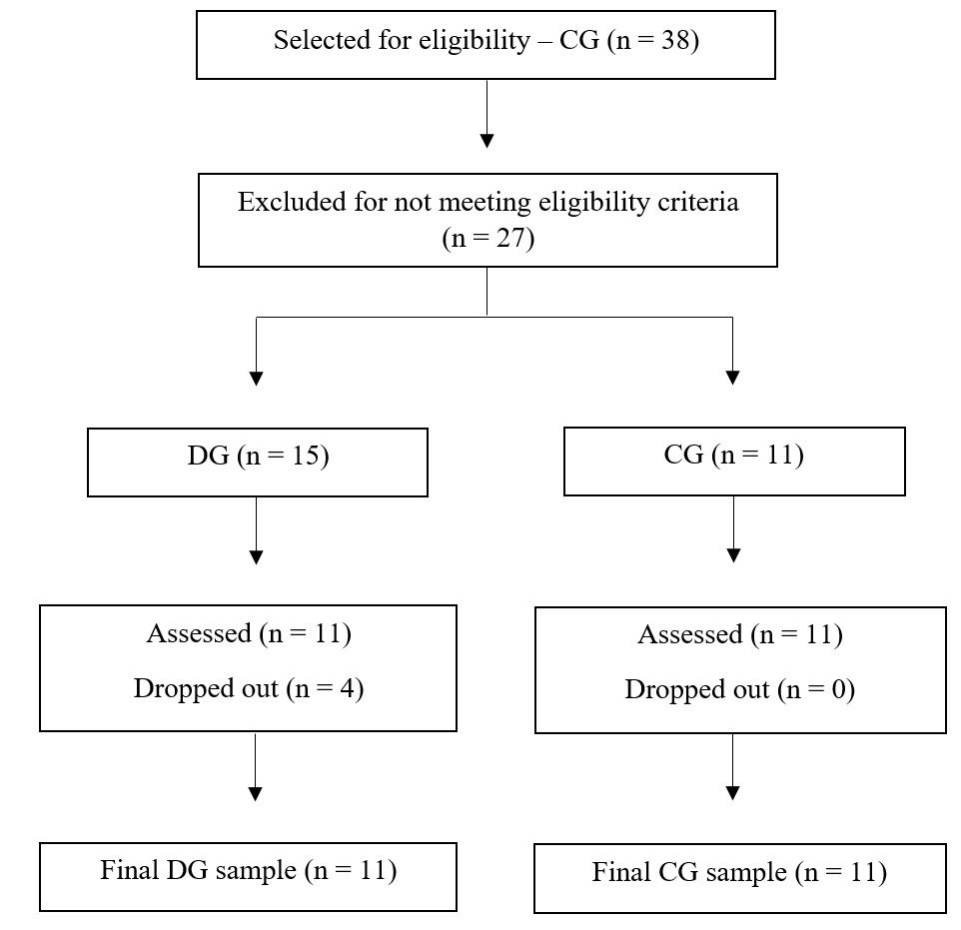
Study flow diagram. CG = control group; DG = detraining group.

The study assessed 22 patients with PAOD, 11 in the DG and 11 in the CG. Table 1 lists data on the characteristics of the sample. It is important to point out that the groups were similar, with no statistically significant differences for age (p = 0.719), ABI (p = 0.455), or sex (p = 0.258). Figure 1 illustrates the process of patient recruitment, selection, and exclusion.

**Table 1:** Characteristics of the samples in both groups.

	DG (n = 11)	CG (n = 11)	p
Body mass (kg)	74±11	75±11	0.839
Height (cm)	167±7	170±9	0.385
BMI	27±4	26±3	0.389
Age (years)	66±8	67±7	0.719
ABI	0.66±0.20	0.73±0.22	0.455
Female sex (%)	5 (45)	2 (18)	0.170
Resting HR (bpm)	76±11	68±12	0.133
Systolic BP (mmHg)	142±25	143±11	0.874
Diastolic BP (mmHg)	76±12	76±6	0.982
Borg	3.27±2	3.64±2	0.676

DG = detraining group; CG = control group; BMI = body mass index; ABI = ankle-brachial index; HR = heart rate; BP = blood pressure; Borg = subjective perceived exertion scale. p < 0.05 indicates statistically significant difference.

**Figure 2:**
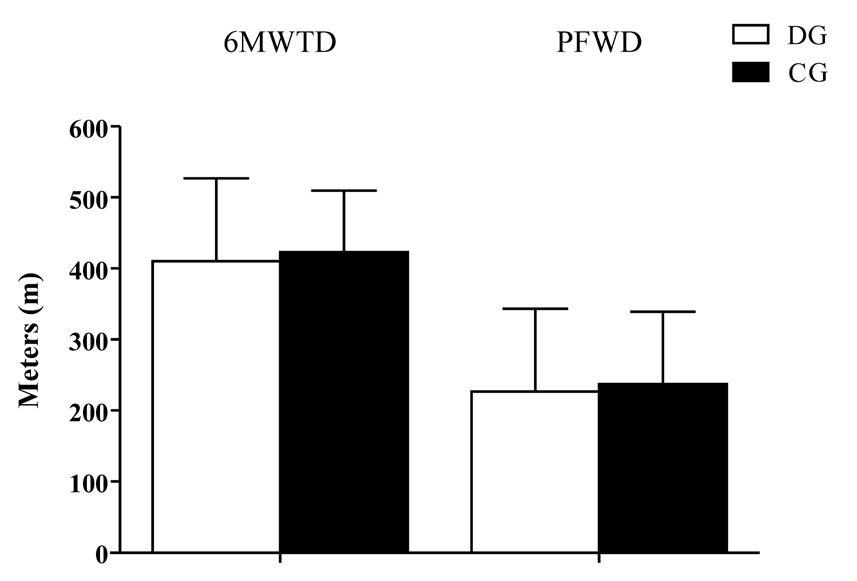
Distance covered in the 6-minute walk test and distance walked free from claudicant pain. 6MWTD = 6-minute walk test distance; PFWD = pain free walk distance; DG = detraining group; CG = control group.

Figure 2 illustrates the main outcomes. There was no statistically significant difference in 6MWTD between the two groups, with mean distances of 388±111 m in the DG and 423±87 m in the CG (p = 0.428). There was also no significant difference in PFWD between the groups, with mean distances of 203±138 m in the DG and 236±104 m in the CG (p = 0.537). The 6MWTD in the DG had been 445 ± 88 meters after the training period; i.e., after detraining there was a mean reduction of 57±119 m (p = 0.145). In turn, the PFWD in the DG had been 214±137 m after the training period; i.e., after detraining there was a mean reduction of 11±37 m (p = 0.341). The mean duration of detraining was 19±10 months.

### Physical activity level

The DG had similar results to the CG for the number of days per week on which the patients engaged in mild walking (DG: 4.55±2.50; CG: 4.36±3.10; p = 0.187). While there was a 68±149 min difference between the DG and CG in the time spent engaged in mild walking, the difference between the groups was not significant (DG: 128±209 min; CG 60±62 min, p = 0.182).

There was no significant difference between the DG and the CG in the number of days per week on which the patients engaged in moderate walking (DG: 4±2.68; CG: 2±1.44; p = 0.055). There was no significant difference between the groups in time engaged in moderate walking, (DG: 76±79 min; CG: 62±72 min, p = 0.669).

The groups had similar results for the number of days per week on which the patients engaged in vigorous walking (DG: 0.82±1; CG: 0.73±1.27; p = 0.858) and in the time in minutes engaged in vigorous walking (DG: 46±76 min; CG: 24±40 min; p = 0.392).

## DISCUSSION

The initial hypothesis that individuals in the DG would exhibit benefits in relation to the CG was based on evidence on the positive effects of detraining on different parameters in other populations.^11^^-^ 13 Additionally, physical training programs promote underlying motor function and neuromuscular repertoire, which is why it was considered that individuals who had undergone detraining could have exhibited superior performance to those who had never engaged in physical training.^4^^,^ 6 ^-^ 8 ^,^ 10 Only McDermott et al.^7^ (in 2019) have investigated the durability of the effects of a supervised physical training program, but this cannot be considered detraining because the participants were monitored remotely by means of phone calls after the in-person physical training program and were encouraged to maintain their exercise routines. Even so, 6 months after the physical training program, these individuals’ 6MWTD and PFWD distances had fallen back to their baseline values.

Even though the present study did not detect any significant differences between the groups, it is important to emphasize that physical training is widely recommended for patients with PAOD, with the objective of promoting increases in functional capacity and, consequently, improving walking performance.^19^ Implementation of remote follow-up strategies appears to be an option for maintenance of the benefits achieved during in-person physical training.^20^ The data observed in the present study underscore the importance of ongoing participation in physical training programs or of planning for after the end of studies with exercise in order to maintain or further improve the outcomes assessed.^21^^,^ 22

The mean difference of 57 m in the DG during the training period compared with after detraining is extremely relevant, since, even after a mean detraining period of 19 months, these individuals did not exhibit significant loss (p = 0.145). Compared with the CG, the DG had a mean difference of 35 m, which was not a significant difference, thereby confirming that the groups were similar. Gardner et al.^23^ demonstrated that a mean difference of 60 m in the 6MWTD is clinically relevant, i.e., trained individuals who undergo detraining have clinically relevant loss. Additionally, individuals who do not participate in training also have impaired functional capacity.

There was also no significant difference in PFWD between the DG and the CG (p = 0.458). From a functional point of view, this is the more important variable for PAOD patients, since reduced PFWD is reflected in reduction in daily activities of living, causing depression and worsening quality of life. A reduction of 30 m is considered clinically relevant in the literature.^23^ Additionally, McDermott^8^ have shown that physical exercise is capable of improving PFWD to a similar extent to revascularization surgery.

The present study has limitations. The first is the small sample size, which is because this is a pilot study. The second is the mean duration of detraining, which was relatively long (19±10 months) and had a large range. This could have the result that the expected effects were not manifest, because of the small sample with considerable heterogeneity. Moreover, the DG, which comprised individuals who had previously participated in a physical training program in a different study, only contained 11 people because four of the 15 people contacted dropped out before undergoing assessments. This is a significant limitation, since the sample size calculation defined the minimum number necessary as 14 individuals in each group; but there was no way of recruiting additional participants to the DG, because there were only 15 in total. One possible strategy for a future study could be to enroll more participants in the CG, with the objective of increasing the sample’s power for statistical inference.

While the study has limitations, it also has strong points. To our knowledge, it is the first study to evaluate the effects of physical detraining on the functional capacity of individuals with PAOD. This is very important to guide clinical practice and find solutions to help patients remain physically active or participating in physical training programs.

This study is very important for the clinical practice of specialists, since it demonstrates that physical detraining provokes significant losses of the adaptations achieved by physical training. It is therefore important that specialists create strategies for patients with PAOD to encourage engagement and continuity in physical training programs.

## CONCLUSIONS

This study assessed physical detraining in individuals with PAOD, comparing two groups. One group participated in a physical training program and then underwent detraining and the other did not take part in a physical training program and did not therefore undergo detraining. No significant differences were detected between these groups in 6MWTD or PFWD. However, it is important to point out that this study’s findings confirm the idea that individuals with PAOD require ongoing attention after participating in a physical training program, including planning for continuation of physical training and engagement in a daily physical activity routine.
