# Supplementary material for: Importance of physical detraining in functional capacity of individuals with chronic peripheral arterial occlusive disease: a cross-sectional pilot study
Source: J Vasc Bras. 2021 May 10;20:e20200237. doi: 10.1590/1677-5449.200237 (PMC8147877; doi:10.1590/1677-5449.200237)
Supplement: Translation

# Translation: Importância do destreinamento físico sobre a capacidade funcional em indivíduos com doença arterial obstrutiva crônica periférica: estudo transversal piloto

**Authors:** Mabel Marciela Ahner, Adamastor Humberto Pereira, Alexandre Araújo Pereira, Gabriel Alves Fonseca, Gabriel Pereira dos Reis Zubaran, Débora dos Santos Macedo, Eduardo Lima Garcia, Leandro Tolfo Franzoni

**Affiliations:** 1 Universidade Federal do Rio Grande do Sul – UFRGS, Hospital de Clínicas de Porto Alegre – HCPA, Porto Alegre, RS, Brasil.; 2 Universidade Federal do Rio Grande do Sul – UFRGS, Hospital de Clínicas de Porto Alegre – HCPA, Ambulatório de Cirurgia Vascular, Porto Alegre, RS, Brasil.; 3 Universidade Federal do Rio Grande do Sul – UFRGS, Programa de Pós-graduação em Ciências da Saúde: Cardiologia e Ciências Cardiovasculares, Porto Alegre, RS, Brasil.

**Keywords:** exercício, doença arterial periférica, claudicação intermitente

## Abstract

**Contexto:**

O treinamento físico é uma estratégia bem estabelecida para a reabilitação da capacidade funcional de indivíduos com doença arterial obstrutiva crônica periférica (DAOP). No entanto, após um programa de treinamento físico, alguns indivíduos podem descontinuá-lo, causando destreinamento. A literatura é escassa sobre os efeitos do destreinamento físico em indivíduos com DAOP; portanto, torna-se importante investigar os efeitos nessa temática.

**Objetivos:**

Avaliar os efeitos do destreinamento físico sobre a capacidade funcional em indivíduos com DAOP.

**Métodos:**

Estudo transversal com 22 indivíduos. Os participantes foram divididos em dois grupos: grupo destreinamento (GD), grupo controle (GC). Foram avaliadas a distância percorrida no teste de caminhada de 6 minutos (DTC6M) e a distância livre de dor claudicante (DLDC), a qual é referida pela distância percorrida até o início da claudicação, ou seja, sem dor.

**Resultados:**

A média da idade foi de 66±8 para o GD e de 67±7 para o GC. Tanto a DTC6M como a DLDC não apresentaram diferenças entre os grupos (p = 0,428 e p = 0,537, respectivamente).

**Conclusões:**

O presente estudo piloto permite concluir que indivíduos com DAOP que participaram de um programa de treinamento físico e posterior destreinamento não tiveram efeitos superiores na sua capacidade funcional em relação a indivíduos que não participaram de programa de treinamento físico. O resultado do presente estudo serve para incentivar a manutenção da prática de exercício físico, visto que o treinamento físico deixa de ser efetivo se ocorrer destreinamento.

## INTRODUÇÃO

A doença arterial obstrutiva crônica periférica (DAOP) reduz a capacidade funcional, em função do processo aterosclerótico das artérias periféricas^1^, com consequente obstrução do fluxo sanguíneo para os membros inferiores, a qual promove um sintoma clássico conhecido como claudicação intermitente^2^. Quanto menor a distância percorrida até apresentar claudicação, ou seja, livre de dor, pior o prognóstico da doença^3^.

O treinamento físico é um dos principais instrumentos para aumentar a capacidade funcional de pessoas com DAOP^4^. A distância percorrida no teste de caminhada de 6 minutos (DTC6M), bem como a distância livre de dor claudicante (DLDC), são importantes desfechos relacionados à capacidade funcional^3^^,^ 5. Diferentes estudos compararam os efeitos do treinamento físico, seja aeróbico, de força, ou combinado, com duração entre 8 e 16 semanas sobre a DTC6M e a DLDC^6^^-^ 10. No entanto, após o tempo de intervenção, geralmente os pacientes descontinuam o programa de treinamento físico.

O destreinamento físico acaba gerando um questionamento sobre a manutenção dos efeitos gerados pelo treinamento físico em relação a DTC6M e DLDC nos pacientes com DAOP. No entanto, há uma lacuna na literatura em termos de estudos que investigam os efeitos do destreinamento físico em DAOP^11^^-^ 13. Diante disso, o objetivo do presente estudo foi comparar os efeitos do destreinamento físico sobre a DTC6M e a DLDC em pacientes com DAOP. A hipótese do presente estudo é que indivíduos que participaram de um programa de treinamento físico e experimentaram destreinamento teriam maiores benefícios quando comparados a indivíduos que não realizaram um programa de treinamento físico.

## MÉTODOS

### Desenho do estudo

Trata-se de um estudo transversal caso-controle. Os participantes foram divididos em dois grupos: grupo destreinamento (GD), no qual foram incluídos indivíduos que participaram de 12 semanas de treinamento físico e tiveram destreinamento de pelo menos 3 meses e máximo de 36 meses; grupo controle (GC), composto por pacientes com DAOP que não participaram de programa de treinamento físico nos últimos 36 meses.

### Local do estudo e participantes

O presente estudo foi realizado no Ambulatório de Cirurgia Vascular de um hospital universitário do sul do Brasil, entre 2018 e 2019. Foram recrutados pacientes acima dos 40 anos com diagnóstico de claudicação intermitente. Além disso, todos os participantes deveriam apresentar índice tornozelo-braço (ITB) abaixo de 0,9. Os critérios de exclusão foram: eventos cardiovasculares que ocorreram menos de 3 meses antes da inclusão; hipertensão grave não controlada (pressão arterial sistólica ≥ 180 mmHg ou pressão arterial diastólica ≥ 110 mmHg) e/ou diabetes não controlada (índice glicêmico ≥ 290); isquemia crítica de membros inferiores; doença pulmonar limitante; e qualquer contraindicação para a realização do teste com exercício.

O estudo foi aprovado pelo comitê de ética em pesquisa local (parecer nº 3.240.172) e conduzido inteiramente de acordo com os padrões éticos estabelecidos na Declaração de Helsinque. Todos os pacientes forneceram consentimento por escrito para participação antes da inscrição. Parte dos pacientes do estudo (GD) fizeram parte de um ensaio clínico randomizado que investigou o efeito de 12 semanas de treinamento físico sobre a capacidade funcional de indivíduos com DAOP.

### Procedimento experimental

#### Capacidade funcional – DT6CM e DLDC

A capacidade de caminhada foi medida pelo teste de caminhada de 6 minutos (TC6M) por um grupo de especialistas, de acordo com as diretrizes do *American Thoracic Society* 14. Durante o TC6M, usamos uma escala visual analógica (EVA)^15^^,^ 16 para medir a dor nos membros inferiores, bem como a escala de sensação subjetiva ao esforço de Borg^17^. O teste foi realizado em um corredor de 30 metros, onde cada paciente foi orientado a caminhar a maior distância possível durante 6 minutos. A pressão arterial e a frequência cardíaca foram medidas antes e depois do teste. Os pacientes foram questionados sobre o início da dor e orientados a caminhar o mais rapidamente possível. A distância percorrida até o início da dor e a distância total foram registradas e expressas em metros.

### Nível de atividade física

No presente estudo, foi utilizado um questionário simples sobre nível de atividade física, baseado no *International Physical Activity Questionnaire* (IPAQ) versão curta^18^. Os pacientes foram arguidos sobre o número de dias da semana, bem como o tempo, em minutos, em que realizaram caminhada leve, moderada e vigorosa.

### Possíveis vieses

Para minimizar possíveis vieses, a amostra foi rigorosamente selecionada, com uma anamnese completa, e com exame clínico realizado pela equipe do Ambulatório de Cirurgia Vascular. Complementarmente, todos indivíduos submetidos ao TC6M foram avaliados pelo mesmo pesquisador, para evitar variabilidade entre avaliadores, mesmo que fosse seguido o protocolo preconizado pela literatura^14^.

### Análise estatística

Para detectar uma diferença de 60 metros (m) para a DTC6 entre os grupos, e 30 m para a DLDC, com desvio padrão igual à diferença a ser detectada para a DTC6, com poder de 80% e nível de significância de 5%, o tamanho da amostra calculado foi de 14 para cada grupo^7^.

Os testes de Shapiro Wilk e Levene foram utilizados para analisar a normalidade e a homogeneidade dos dados, respectivamente. Para analisar as diferenças entre os grupos, foram utilizados o teste *t* de Student (não pareado) ou o teste de Mann-Whitney U para os desfechos principais (DTC6M e DLDC) e demais variáveis contínuas, e o teste de qui-quadrado para as variáveis categóricas. Os dados foram apresentados por meio de médias e desvio padrão caso apresentassem distribuição normal, ou mediana e intervalo interquartil, caso contrário. Os dados foram analisados por meio do *software* SPSS verão 20.0 (IBM, Corporation, Armonk, NY, EUA). Os valores de p < 0,05 foram considerados estatisticamente significativos.

## RESULTADOS

O presente estudo avaliou 22 pacientes com DAOP, sendo que 11 faziam parte do GD e 11 do GC. Os dados de caracterização da amostra estão expostos na Tabela 1. Importante destacar que os grupos foram semelhantes, sem apresentar diferenças estatisticamente significativas para idade (p = 0,719), ITB (p = 0,455) e gênero (p = 0,258). A Figura 1 expressa o processo de recrutamento e seleção dos participantes, bem como de exclusão.

**Tabela 1:** Características da amostra para ambos os grupos.

	GD (n = 11)	GC (n = 11)	p
Massa corporal (kg)	74±11	75±11	0,839
Altura (cm)	167±7	170±9	0,385
IMC	27±4	26±3	0,389
Idade (anos)	66±8	67±7	0,719
ITB	0,66±0,20	0,73±0,22	0,455
Gênero feminino (%)	5 (45)	2 (18)	0,170
FC repouso (bpm)	76±11	68±12	0,133
PA sistólica (mmHg)	142±25	143±11	0,874
PA diastólica (mmHg)	76±12	76±6	0,982
Borg	3,27±2	3,64±2	0,676

GD = grupo destreinamento; GC = grupo controle; IMC = índice de massa corporal; ITB = índice tornozelo-braço; FC = frequência cardíaca; PA = pressão arterial; Borg = escala de sensação subjetiva ao esforço. p < 0,05 indica diferença estatisticamente significativa.

**Figura 1:** Fluxograma do estudo. GC = grupo controle; GD = grupo destreinamento.

Os desfechos principais estão expressos na Figura 2. A DTC6M não apresentou diferença estatisticamente significativa entre os dois grupos, com valor médio de 388±111 m para o GD e de 423±87 m para o GC (p = 0,428). A DLDC também não apresentou diferença significativa entre os grupos, com um valor médio de 203±138 m para o GD e de 236±104 m para o GC (p = 0,537). O valor da DTC6M para o GD após o período de treinamento foi de 445 ± 88 metros, ou seja, após destreinamento houve uma redução média de 57±119 m (p = 0,145). O valor de DLDC para o GD após o período de treinamento foi de 214±137 m, ou seja, após destreinamento houve redução média de 11±37 m (p = 0,341). O tempo médio de destreinamento foi de 19±10 meses.

**Figura 2:** Distância percorrida no teste de caminhada de 6 minutos e distância livre de dor claudicante. DTC6M = distância percorrida no teste de caminhada de 6 minutos; DLDC = distância livre de dor claudicante; GD = grupo destreinamento; GC = grupo controle.

### Nível de atividade física

O GD apresentou resultados semelhantes, comparado ao GC, quanto ao número de dias da semana em que os pacientes praticavam caminhada leve (GD: 4,55±2,50; GC: 4,36±3,10; p = 0,187). Apesar da diferença de 68±149 min entre o GD e o GC em relação ao tempo praticado de caminhada leve, os grupos não apresentaram diferença significativa (GD: 128±209 min; GC 60±62 min, p = 0,182).

O GD não apresentou diferença significativa, comparado ao GC, quanto ao número de dias da semana em que os pacientes praticavam caminhada moderada (GD: 4±2,68; GC: 2±1,44; p = 0,055). Em relação ao tempo praticado de caminhada moderada, os grupos não apresentaram diferença significativa (GD: 76±79 min; GC: 62±72 min, p = 0,669).

Ambos os grupos apresentaram resultados semelhantes quanto ao número de dias da semana em que os pacientes praticavam caminhada vigorosa (GD: 0,82±1; GC: 0,73±1,27; p = 0,858), e também quanto ao tempo de caminhada vigorosa em minutos (GD: 46±76 min; GC: 24±40 min; p = 0,392).

## DISCUSSÃO

A hipótese inicial de que os indivíduos do GD apresentariam benefícios em relação ao GC se dá em função de algumas evidências a respeito dos efeitos positivos do destreinamento sobre diferentes parâmetros em outras populações^11^^-^ 13. Além disso, um programa de treinamento físico proporciona base motora e repertório neuromuscular, razão pela qual os indivíduos submetidos a destreinamento podem apresentar desempenho superior comparados àqueles que nunca realizaram treinamento físico^4^^,^ 6 ^-^ 8 ^,^ 10. Apenas McDermott et al.^7^, em 2019, investigaram a durabilidade dos efeitos de um programa de treinamento físico supervisionado, mas aqui não se pode considerar destreinamento, pois os indivíduos eram monitorados à distância ao final do programa presencial de treinamento físico, por meio de ligações, e encorajados a manter a rotina de exercícios. Mesmo assim, 6 meses após o treinamento físico, os indivíduos reduziram valores de DTC6M e DLDC, retomando os valores basais.

Mesmo que o presente estudo não tenha apresentado diferenças significativas entre os grupos, é importante destacar que o treinamento físico é amplamente recomendado para pacientes com DAOP, com a finalidade de promover aumento da capacidade funcional e, consequentemente, melhorar a performance da marcha^19^. A implementação de estratégias de seguimento à distância parece ser uma alternativa para a manutenção dos benefícios adquiridos durante o treinamento físico presencial^20^. Os dados encontrados no presente estudo reforçam que a permanência em programas de treinamento físico ou o planejamento após o término de estudos com exercício são importantes para se manter, ou melhorar ainda mais, os desfechos avaliados^21^^,^ 22.

A diferença média de 57 m para o GD em relação ao período de treinamento comparado ao destreinamento é extremamente relevante, pois, mesmo após um período médio de destreinamento de 19 meses, os indivíduos não apresentaram perda significativa (p = 0,145). Em comparação ao GC, o GD possui uma diferença média de 35 m, sem diferença significativa, comprovando assim que os grupos eram semelhantes. Gardner et al.^23^ demonstraram que uma diferença média de 60 m para a DTC6M é clinicamente relevante, ou seja, indivíduos treinados que sofrem destreinamento têm uma perda clinicamente relevante. Além disso, indivíduos que não realizam treinamento também apresentam prejuízo na capacidade funcional.

A DLDC também não apresentou diferenças significativas entre o GD e o GC (p = 0,458). Essa é a variável mais importante para a DAOP, do ponto de vista funcional, pois reduzir a DLDC gera redução nas atividades de vida diária, causando depressão e piorando a qualidade de vida. Uma redução clinicamente relevante de 30 m é considerada pela literatura^23^. Além disso, McDermott^8^ demonstraram que o exercício físico é capaz de melhorar a DLDC, assim como a cirurgia de revascularização.

O presente estudo possui limitações: a primeira é o pequeno tamanho da amostra, por se tratar de um estudo piloto; a segunda é o tempo médio de destreinamento, o qual foi relativamente grande (19±10 meses), com uma variabilidade alta. Isso pode fazer com que os efeitos esperados não se expressem, por tratar-se de uma pequena amostra com grande heterogeneidade. Além disso, o GD, o qual participou previamente de um programa de treinamento físico em outro estudo, foi composto por 11 indivíduos, visto que, dos 15 contatados, quatro desistiram de fazer as avaliações. Isso é uma grande limitação, pois o cálculo amostral previu um número necessário de 14 indivíduos para cada grupo; no entanto, não tivemos como incluir mais participantes no GD, visto que tínhamos apenas 15 no total. Uma possível estratégia para um estudo futuro será incluir mais participantes no GC, com a finalidade de aumentar o poder da amostra de inferência estatística.

Apesar de o estudo possuir limitações, também possui méritos. Segundo nosso conhecimento, este é o primeiro estudo que avaliou os efeitos do destreinamento físico sobre a capacidade funcional de indivíduos com DAOP. Isso é muito importante para guiar a prática clínica e encontrar alternativas para que os pacientes se mantenham fisicamente ativos ou inseridos em programas de treinamento físico.

O presente estudo é fundamental para a prática clínica do especialista, ao demonstrar que o destreinamento físico promove perdas significativas nas adaptações geradas pelo treinamento físico. Portanto, é importante que o especialista crie estratégias de engajamento e continuidade em programas de treinamento físico para os pacientes com DAOP.

## CONCLUSÃO

O presente estudo avaliou o destreinamento físico em indivíduos com DAOP comparando dois grupos, um que participou de um programa de treinamento físico e realizou destreinamento e outro que não participou de um programa de treinamento físico e, consequentemente, não realizou destreinamento. Não foram encontradas diferenças significativas entre os grupos para a DTC6M e a DLDC. Apesar disso, é importante ressaltar que os achados do presente estudo reforçam a ideia de que, após a participação em um programa de treinamento físico, os indivíduos com DAOP merecem atenção, desde o planejamento para a continuação do treinamento físico até o engajamento em uma rotina de atividade física diária.
